# Plasticity towards Rigidity: A Macrophage Conundrum in Pulmonary Fibrosis

**DOI:** 10.3390/ijms231911443

**Published:** 2022-09-28

**Authors:** Ezgi Sari, Chao He, Camilla Margaroli

**Affiliations:** 1 Department of Medicine, Division of Pulmonary, Allergy & Critical Care Medicine, University of Alabama at Birmingham, Birmingham, AL 35294, USA; 2 Department of Pathology, Division of Cellular and Molecular Pathology, University of Alabama at Birmingham, Birmingham, AL 35294, USA

**Keywords:** idiopathic pulmonary fibrosis, activated macrophages, fibrotic macrophages, EMT

## Abstract

Idiopathic pulmonary fibrosis (IPF) is a progressive, chronic, and ultimately fatal diffuse parenchymal lung disease. The molecular mechanisms of fibrosis in IPF patients are not fully understood and there is a lack of effective treatments. For decades, different types of drugs such as immunosuppressants and antioxidants have been tested, usually with unsuccessful results. Although two antifibrotic drugs (Nintedanib and Pirfenidone) are approved and used for the treatment of IPF, side effects are common, and they only slow down disease progression without improving patients’ survival. Macrophages are central to lung homeostasis, wound healing, and injury. Depending on the stimulus in the microenvironment, macrophages may contribute to fibrosis, but also, they may play a role in the amelioration of fibrosis. In this review, we explore the role of macrophages in IPF in relation to the fibrotic processes, epithelial–mesenchymal transition (EMT), and their crosstalk with resident and recruited cells and we emphasized the importance of macrophages in finding new treatments.

## 1. Introduction

Idiopathic pulmonary fibrosis (IPF) is a progressive, chronic, and ultimately fatal diffuse parenchymal lung disease. It is the most common interstitial lung disease (ILD) affecting mostly the subpleural parenchyma. IPF is characterized by irregular fibrotic patches, fibroblastic foci, and honeycomb changes [1,2,3]. The mortality rate in IPF approaches 50% within 3–5 years after the initial diagnosis [4,5], with survival rates lower than in some cancer patients [6]. In spite of critical scientific advances, the current metrics for diagnosis and treatment (published in 2022), are still not sensitive enough to identify patients with IPF in the early stage of the disease, which contributes to its poor prognosis [7].

**Figure 1:**
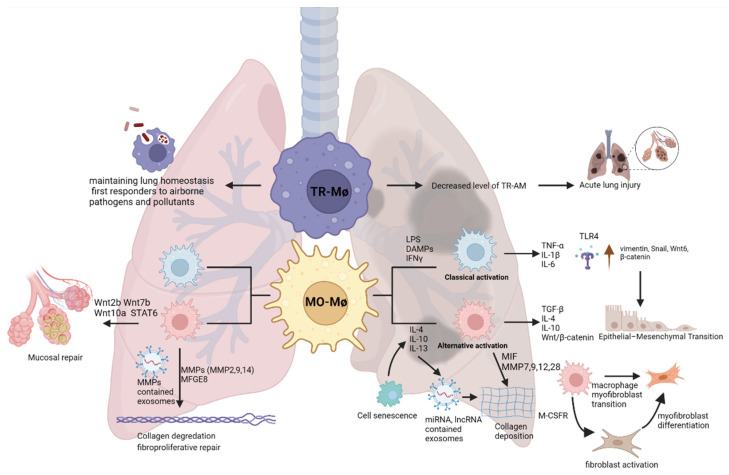
Schematic representation for hypothetical the role of alveolar macrophages in pulmonary fibrosis. The left side of the figure represents the how macrophages can clean the fibrotic foci and keep homeostasis, the right side shows macrophage-induced fibrosis.

While the molecular mechanisms of fibrosis in IPF patients are not fully understood, it is believed that repeated injuries to the alveolar epithelium may lead to dysregulated repair mechanisms and aberrant deposition of extracellular matrix [8,9]. Following the injury in the alveolar epithelium, recruited macrophages can contribute to the pathogenesis of pulmonary fibrosis via the secretion of a plethora of mediators such as cytokines, interleukins, transforming growth factor beta (TGF-β), connective tissue growth factor (CTGF), epidermal growth factor receptor (EGFR) ligands, and proteases [10,11,12]. Here, we review how molecular mechanisms of macrophage plasticity may orchestrate the onset of IPF and contribute to its progression (Figure 1), as well as current therapeutics and potential future macrophage-directed therapies.

## 2. Development of Pharmacological Treatment for IPF

Liebow et al. first described the histological feature of the usual interstitial pneumonia (UIP) pattern: coexisting fibrosis and interstitial/airway inflammation [13]. For decades, IPF has been considered as a chronic inflammatory condition in which repeated injuries lead to chronic scaring/fibrosis, where excessive oxidative stress due to chronic inflammation plays a key role in the pathogenesis. Hence, steroids have been the cornerstone for maintenance therapy as early data showed that high-dose steroids led to clinical improvement in a small population of patients (<30%) diagnosed with IPF [14]. Subsequently, Raghu et al. examined whether adding azathioprine, another potent immunosuppressive medication, to prednisone could benefit patients with IPF [15]. In this small (27 patients), double-blinded, randomized, placebo-controlled trial, prednisone and azathioprine combination therapy trended towards improved survival and reduced rate of decline in lung function measured by pulmonary function test (PFT). However, none of these parameters reached statistical significance. In addition to the escalation of immunosuppressive therapy, researchers have evaluated the efficacy of combining antioxidants with immunosuppressive medications. Behr et al. conducted an open-label trial with high-dose N-acetylcysteine (NAC) in 18 patients with or without immunosuppressive medications [16]. They found that 12 weeks of high-dose NAC treatment improved lung function index, a composite endpoint incorporating several PFT parameters. Moreover, patients who benefited most from NAC therapy were those on immunosuppressive medications. The follow-up double-blinded, randomized, placebo-controlled Idiopathic Pulmonary Fibrosis International Group Exploring N-Acetylcysteine I Annual (IFIGENIA) trial examined the addition of NAC or placebo to prednisone and azathioprine (the standard care arm) and found that adding NAC to prednisone/azathioprine slowed the decline of lung function. However, no survival benefits were observed [17]. The encouraging data from the IFIGENIA trial led to the NIH-sponsored double-blinded, randomized, placebo-controlled Prednisone, Azathioprine, and N-Acetylcysteine: A Study That Evaluates Response (PANTHER) trial [18]. In PANTHER, investigators evaluated the benefits of a combined triple therapy of prednisone, NAC, and azathioprine, compared with placebo. The trial was terminated earlier due to increasing mortality and rate of acute exacerbation in the experimental group compared with the placebo arm.

The failure of the PANTHER trial, together with two failed randomized Phase III trials evaluating interferon gamma-1β [19,20], marked a significant shift in developing therapies for IPF. Advances in basic and translational science into the molecular mechanism of IPF showed that IPF is a complex disease condition that begins with repeated epithelial injury and leads to recruitment of pro-fibrotic macrophages and activation of (myo)fibroblasts. Macrophages involved in the pathogenesis of IPF are no longer considered solely pro-inflammatory, but profibrotic and generate multiple growth factors. Consequently, there has been significant interest in developing anti-fibrotic, rather than anti-inflammatory, therapeutics targeting growth factor such as TGF-β. The subsequent double-blinded, randomized, placebo-controlled trials examining the two novel anti-fibrotics, namely pirfenidone and nintedanib, demonstrated a significantly reduced decline in lung function compared to placebo for both drugs [21,22,23]. FDA approved both medications for the treatment of IPF in 2014, and both received conditional recommendation from ATS/ERS practice guidelines in 2015 [24]. The exact target of pirfenidone remains unclear [25]. Initially developed as an anti-inflammatory medication, it was found to affect fibrosis development in various animal models. Nintedanib, a tyrosine kinase inhibitor, was initially designed for cancer treatment. It targets multiple growth factor signaling pathways such as fibroblast growth factor receptor-1 (FGF-1), vascular endothelial growth factor receptor-2 (VEGFR-2), and platelet-derived growth factor receptor (PDGF-R) α and β [26]. More recently, nintedanib has been approved to be used to treat systemic sclerosis-associated interstitial lung disease (SENSCIS) and progressive fibrosing interstitial lung disease (INBUILD) [27,28,29]. The effectiveness of anti-fibrotics in these non-IPF conditions highlights the shared profibrotic pathways in various ILDs and targeting profibrotic mediators can modulate ILD progression.

## 3. Macrophages in Fibrosis

Macrophages are central to lung homeostasis as well as in the orchestration of the immune response following an insult [30,31,32,33], which makes them an attractive target for future therapies. Therefore, here we will explore their role in IPF in relation to the fibrotic processes, epithelial–mesenchymal transition (EMT), and their crosstalk with resident and recruited cells.

Macrophages are an important part of host defense, and they are at the interface between innate and adaptive immunity [34]. Macrophages constitute one of the first lines of defense against external pathogens and insults [35], and are crucial for the phagocytic clearance of microorganisms, apoptotic cells, and cancer cells [36,37,38]. Over the decades it has been thought that the recruitment and activation of inflammatory cells contribute to pulmonary fibrosis. Indeed, recent studies have shown that the disturbed wound healing process and the activation of fibrotic responses can increase the activation and polarization of macrophages and lymphocytes [39]. It is also reported that IPF patients have increased levels of alveolar macrophages [32], making these cells a crucial player in fibrosis.

There are two distinct groups of macrophages in the lungs: interstitial macrophages (IMs) and alveolar macrophages (AMs) [40,41,42]. IMs are located within the lung parenchymal tissue, and they have regulatory functions including, tissue remodeling and maintenance of homeostasis. AMs are present in the alveoli, and they are the most abundant resident immune cells in lung homeostasis. They are involved in the phagocytosis of the external particles and maintain the surfactant catabolism in the alveoli [43,44,45,46,47,48]. Although the ontology of lung macrophages is still debated, it is thought that macrophages in the lung have three different developmental waves in the mouse lung tissue [49]. The first wave of macrophages is developed from yolk sac precursors, and they spread throughout the lung interstitium during embryonic development. In the second wave, the fetal liver-derived macrophages migrate to the alveoli and become alveolar macrophages. Third-wave macrophages are derived from bone marrow and reside in the lung interstitium during homeostasis [49]. According to their activation status, macrophages can be divided into two major subsets: classical activation (M1, pro-inflammatory/cytotoxic) and alternative activation (M2, anti-inflammatory/wound repair) [50,51]. Both populations can be further subdivided based on their function and gene expression: M2a works on phagocytosis, M2b is responsible for immunoregulation, and M2c plays a role in tissue modification and matrix deposition [50,52]. While these subsets are very well defined in the mouse, the dichotomy between the different macrophage phenotypes becomes less clear in the human lung. Nevertheless, their polarization states highlight the plastic potential of these cells.

Since macrophages are one of the main regulators of the immune response, their activation status might influence other cells’ behavior [53]. In IPF, it has been suggested that the microenvironment during injury may affect how monocytes differentiate into alveolar macrophages and promote tissue damage or fibrosis [54]. Indeed, several studies showed that monocytes can be drawn to the lung, where they can differentiate into Monocyte-derived AMs (Mo-AMs) and be alternatively activated towards the profibrotic or “M2” phenotype [55,56,57]. 

In IPF, CD163^+^ M2 macrophages are enriched in the fibrotic areas of the human lung [58]. Single cell analysis showed that tissue-resident alveolar macrophages, tissue-resident peribronchial and perivascular interstitial macrophages, and monocyte-derived alveolar macrophages are located in the fibroblastic foci. Further, single cell RNA sequencing revealed the presence of a Mo-AM population with pro-fibrotic gene signatures in an in vivo model of lung fibrosis. Presence of these Mo-AMs was demonstrated to be dependent upon signaling via the macrophage colony-stimulating factor receptor (M-CSFR), as the absence of M-CSFR depleted their presence in the fibrotic niches and ameliorated the pathology [59]. 

In the bleomycin-induced pulmonary fibrosis model, inflammatory macrophages increase immediately, reach the peak level on day 3, and slowly reduce until day 21. M1-like alveolar macrophages are the major population in the bronchoalveolar lavage (BAL) fluid at a steady state, however, after bleomycin exposure, M2-like alveolar macrophages gradually increased and reached the maximum level on day 14 which correlated with collagen deposition [31,60]. In an elegant study, Misharin and colleagues further highlighted the importance of Mo-AMs in the development of fibrosis. With a series of experiments using genetically engineered in vivo models, they were able to discern the role of Mo-AMs in a model of lung fibrosis. Briefly, Mo-AMs, but not AMs, contributed to the development of lung fibrosis in response to bleomycin and TGF-β via expression of pro-fibrotic genes, as necroptosis of Mo-AMs, attenuated bleomycin-induced lung fibrosis [54]. Further, they validated the expression of those genes in the human lung, showing the translational implication of the in vivo findings. Lastly, they showed that Mo-AMs persisted in the lung for a year after the initial fibrotic insult, highlighting a potential pathological mechanisms that could lead to aberrant responses to future insults.

In other models of fibrosis relevant to IPF’s risk factors, macrophages played a major role in the fibrotic processes after herpesvirus infection [61], and during radiation-induced lung fibrosis (RIF) [58]. Overall, in fibrosis macrophages play a pivotal role in the orchestration of the profibrotic response. Interactions between macrophages and other immune cells [62], as well between macrophages and parenchymal and mesenchymal lung cells, such as fibroblasts and epithelial cells, coupled with the degradation of the extracellular matrix (ECM) [63,64,65], contribute to fibrogenesis.

### 3.1. Cytokines, TGF-β and Wnt/β-Catenin Signaling

Macrophage polarization depends on the microenvironment. Toll-like receptor 4 (TLR4) signaling through Myd88-dependent IRAK-M expression has been shown to be increased in peripheral blood cells from idiopathic pulmonary fibrosis patients compared to controls and was linked to the alternative macrophage activation, profibrotic phenotype, and collagen production [66,67]. Further, it has also been reported that TLR signaling could trigger the pathology of pulmonary fibrosis by increasing the production and release of cytokines such as IL-6, TNF-α, and IL-1β [68]. In IPF, patients display higher levels of IL-6 in plasma and alternatively activated macrophages [31,69,70]. Upon TLR4 stimulation, alveolar macrophages start to release IL-6, IL-1β, and TNF-α, which trigger alveolar type 2 cells to secrete IL-1β and TNF-α helping recruit more leukocytes to the scar area [71], therefore fueling the inflammatory processes linked to fibrosis. While the ligand(s) responsible for TLR4 activation remain poorly defined in IPF, it will likely be a scenario where a combination of Pathogen-Associated Molecular Patterns (PAMPs) and Damage-Associated Molecular Patterns (DAMPs) will be required for the increased TLR4-mediated signaling [67,72,73,74,75,76].

Of the other cytokines potentially involved in IPF, IL-4, IL-10, and IL-13 represent another pathological loop that could fuel the fibrotic process. IL-10 expression has been linked to an increase in the T helper type 2 response, which modulates the production of the profibrotic cytokines IL-4 and IL-13 [77]. In the post-irradiation lung fibrosis model, IL-4 production by macrophages exacerbated the fibrotic process [78], while in the bleomycin model, blockade of IL-4 reduced fibrosis. In another model of IPF, IL-4 mediated the phosphorylation of STAT6, which regulated the M2 polarization via a redox-dependent mechanism, independent of Th2, and contributed to fibrosis [79,80]. Further, IL-13 contributes to fibrosis by changing the extracellular matrix (ECM) structure via the activation of ECM-degrading enzymes and promoting the fibrotic signaling pathways [81,82,83]. Although macrophages promote the wound healing processes by this signaling cascade [10], prolonged cytokine production deteriorates epithelial injury and promotes fibrosis. Long term inflammation might affect fibrosis like a major fibrotic process mediator TGF-β and modulating cytokines production from macrophages might be useful to ameliorate the pathology. 

TGF-β is a multifunctional regulatory protein acting on the SMAD family signaling [84,85]. It plays a role in a plethora of processes, including wound healing, inflammation, EMT, myofibroblast activation, collagen deposition, and apoptosis, and it is a major stimulator of fibrosis [33,86,87,88,89,90,91,92]. TGF-β is released by activated fibroblasts, platelets, and lymphocytes, including macrophages [90,93,94,95]. At homeostasis, it modulates macrophage differentiation in an autocrine fashion [33] via TGF-βR signaling, which is upregulated by PPAR-γ. However, in inflammatory conditions it can polarize macrophages towards an alternative activation state in an autocrine manner [95,96]. In human alveolar macrophages, stimulation with IL-4 and/or IL-10 increased the activation of STAT3, which led to the production of TGF-β [70]. Likewise, IL-13 increases TGF-β production by macrophages via IL-3Rα and AP-1-mediated activation of the TGF-β promoter [81]. TGF-β production is also regulated by the Wnt signaling pathway, one of the essential triggers of pulmonary fibrosis, and a major regulator of epithelial cell fate during development and injury [97,98]. In the bleomycin-mediated pulmonary fibrosis, deletion of the Wnt signaling pathway co-receptor LPR5, reduced TGF-β production AT2 cells and macrophages [99]. 

In the IL-4-treated M2 macrophages and epithelial cell co-culture system, TGF-β was increased in epithelial cells and EMT was triggered by TGF-β /Smad2 signaling pathway [100]. Interestingly, Murray et al. showed that depletion of M2 macrophages decreased collagen levels and attenuated fibrosis even in the presence of TGF-β [101], suggesting an important role for macrophage-derived IL-13, as highlighted by Borthwick and colleagues [102]. Indeed, in another study, the fibrosis regressed in IL-13^−/−^ mouse group even though TGF-β levels were same as in the wild type controls [93]. Overall, macrophage-derived TGF-β and IL-13 may contribute to chronic fibrosis in a two-hit model and affect the polarization of other macrophages, as well as key fibrotic mechanism such as EMT, myofibroblast differentiation, collagen deposition, and re-vascularization [31,103,104]. 

### 3.2. Role of Macrophages in Fibrogenesis, Myofibroblasts Differentiation and Epithelial–Mesenchymal Transition 

EMT is a tissue remodeling process in which epithelial cells lose their junctional structures (such as E-cadherin, and ZO-1) and gain distinct components of mesenchymal cell proteins and remodel the ECM (α-SMA, N-cadherin, and Vimentin) [105,106,107]. EMT contributes to fibrosis, invasion, and tumor metastasis [107,108]. In the late stage of injury, recruited macrophages by the extracellular microenvironment contribute to EMT [109,110,111]. Classical activation of macrophages and the release of its associated inflammatory factors increases EMT-related transcription factor levels, such as NF-κB, Snail, and Slug, and their nuclear localization in epithelial cells [110].

In co-culture experiments with classically or alternative-activated macrophages and epithelial or mesothelial cells, the levels of E-cadherin were downregulated, while α-SMA was upregulated. IL-8 increased significantly in the macrophage co-culture system and N-Cadherin and vimentin expression was elevated by JAK2/STAT3/Snail signaling pathway [112]. Moreover, epithelial cells underwent EMT, and concomitant further polarization to M2 was observed, suggesting two-way crosstalk between the epithelium and the macrophages that could worsen the pathology of IPF [64,100,113,114]. In cancer, CD68^+^ macrophages located around the tumor core contributed to the EMT process by triggering the downregulation of E-cadherin and the upregulation of vimentin, N-cadherin, and Snail in the epithelial and cancer cells via the release of TGF-β [112,115,116], a process potentially present in IPF as well. In M1 macrophages and mesothelial cells co-culture, the TRIF-dependent TLR4 signaling pathway was activated in the mesothelial cells to produce α-SMA and cells underwent the EMT process [114]. Overall, these studies show that inflammatory and pro-fibrotic cytokines released by activated macrophages in the microenvironment, induce the expression of EMT markers. Understanding the effect of activated macrophages on other cells in the fibrotic niche is an important area of research, as it could impact not only the development of IPF, but also the long-term inflammation-related progression of fibrosis. Further, focusing on the macrophage-related EMT process and modulating the cross-talk between macrophages and other cells in the niche might be a critical component of future therapeutics.

Activated macrophages promote fibrogenesis by releasing growth factors and triggering the proliferation and collagen synthesis of fibroblasts [117,118]. Single-cell RNA sequencing experiments in lung tissue from patients with fibrotic lungs showed co-expression of monocyte/macrophage and myofibroblast markers [119]. Dysregulated wound healing processes and aberrant cross-talks between macrophages and myofibroblasts may accelerate the progression of fibrosis. The crosstalk between these two cell types promotes myofibroblast activation resulting in fibrosis [10,120], while the accumulation of ECM components mediated by myofibroblasts increases the recruitment of macrophages [65]. This process is partly mediated by Cadherin-11 (CDH11), an adhesion protein. After adhesion via CDH11, macrophages can produce TGF-β and trigger myofibroblast differentiation in the fibrotic lung tissue in mice and humans [121]. In the macrophage-fibroblast co-culture with LPS-activated macrophages, fibroblasts secrete more matrix metalloprotease 7 (MMP7) and TNF-α; while during the co-culture with IL-4 activated macrophages fibroblasts express collagen, and TGF-β [118], thus promoting the pathological progression of the disease. Further, it has been reported that M2 macrophages influence myofibroblast differentiation and increase α-SMA levels by activation of the Wnt/β-catenin signaling pathway [31,122]. IL-4/IL-13 increases the phosphorylated STAT6 and JAK1 and up-regulates FIZZ1, which is a protein responsible for myofibroblast differentiation [123]. 

Although data are limited in IPF, it has been shown that bone marrow-derived macrophages can also increase the number of myofibroblasts via macrophage to myofibroblast transition (MMT), as reviewed in [124]. In the unilateral ureteral obstruction-induced pulmonary fibrosis model, 30% of myofibroblasts were CD68^+^ and α-SMA^+^, and alternatively activated macrophages were shown to directly transdifferentiate into collagen-producing α-SMA^+^ myofibroblasts via TGF-β/Smad3 signaling [113,119,125,126,127,128]. MTT also was also observed via STAT1, STAT3, and NF-ĸB signaling pathways in lung fibrosis [119], suggesting the cooperation of several signaling pathways. A potential mechanism of action for MMT has been elucidated in the bleomycin and TGF-β induced pulmonary fibrosis in vivo models, where the lung myofibroblasts induced epigenetic modifications in the tissue macrophages via histone lactylation, skewing the polarization of the alveolar macrophages into the pro-fibrotic phenotype [129]. Altogether, these studies indicate that macrophages can both stimulate fibroblast-myofibroblast differentiation, as well as potentially contribute to the pathology via MTT. 

### 3.3. Role of Macrophages on ECM

The ECM microenvironment controls homeostasis and regulates cell adhesion, migration, cell cycle, metabolism, and cell differentiation [130]. ECM degradation occurs during fibrosis by matrix metalloproteases (MMPs) and other enzymes [131,132]. Dysregulation of the deposition and degradation of ECM promotes the formation of the fibrotic niches [133], and targeting the pathways regulating ECM degradation and collagen degeneration in fibrosis could be a promising treatment [134].

The effect of macrophages on ECM is a double-edged sword. Macrophage-derived MMPs exert antagonistic roles in fibrosis with regard to the regulation of the ECM and the progression of IPF, reviewed in [133]. In the bleomycin-induced pulmonary fibrosis model, TGF-β1 was overexpressed by macrophages and mesenchymal cells and ECM deposition occurred [94]. Overexpressed TGF-β1 can increase the ECM deposition by reducing the ECM degradation through reduction the antifibrotic MMP1 and induction of TIMP1 (MMP inhibitor) [135]. On the other hand, a fibrotic active-MMP7 is abundantly increased in IPF patients, and it is localized on the surface of alveolar macrophages [136]. Further, MMP28 contributes to pulmonary fibrosis and M2 polarization in the lung [137,138]. In the late stage of injury, macrophages overexpress MMP-9, which contributes to EMT via osteopontin cleavage [109]. MMP12, a macrophage-secreted elastase, is highly induced in the lung in response to injury and regulates fibrosis by controlling the expression of MMP-2 and MMP-13 in IL-13Rα-dependent manner. The loss of MMP-12 increased the level of MMP-2 and MMP-13 and ameliorated fibrosis [138]. Further, macrophage-released MMP-14 and MMP-9 can also clear the fibrotic area in the tissue by breakdown the extracellular matrix and digesting the collagen [104,134], while milk fat globule EGF 8 (MFGE8)-positive macrophages can phagocytose the collagen and attenuate fibrosis [139]. Depending on the status of the signals in the ECM, macrophages may contribute to fibrosis by secreting MMPs that increase TGF-β release in a fibrotic environment, or they may play a role in the clearance of fibrosis by taking part in collagen degradation. Clarifying the underlying signals and molecular mechanisms of such dichotomy may prove to be critical for the development of effective therapies.

### 3.4. Macrophage-Derived Extracellular Vesicles 

Exosomes are small (30–150 nm) membrane vesicles and are released into the extracellular environment [140]. Exosomes and extracellular vesicles (EVs) carry RNA, including microRNA (miRNA), transfer RNA (tRNA), ribosomal RNA (rRNA), messenger RNA (mRNA), and additional structural and non-coding RNAs, as well as effector proteins. They regulate cell–cell signaling [141,142]. EV-carried miRNA can contribute to fibrosis [143,144,145] as highlighted by Let-7 miRNA. Let-7 targets IGF1, which is overexpressed in alveolar macrophages and can stimulate collagen production in IPF [146,147]. Further, in macrophages, Let-7 can change the M1-M2 polarization in the fibrotic lung [148]. IL-4 and IL-13 upregulate miR-142-5p and downregulate miR-130a-3p in macrophages so that they can sustain the profibrogenic effect [149]. As Kishore and Petrek discussed in their review, macrophages can join the EMT and ECM deposition by regulating fibrotic signaling pathways via miRNA upregulation/downregulation [150]. The polarization of M1 alveolar macrophages by TGF-β1 reduced the level of miR-124 and over-expression of miR-124 suppressed M1 alveolar macrophage polarization [151]. Macrophage-Derived miR-21-5p-containing exosomes increased the α-SMA, TGF-β1, and collagen accumulation in the resident cells in Smad7 dependent manner and contributed to the fibrosis [152].

EVs also showcase proteolytic enzymes on their lipid capsules, which can cleave surface proteins and degrade the ECM [153,154]. MMP14+ EVs degrade collagen, fibronectin, and vitronectin in the ECM and activate MMP-2, which is another collagen-degrading protease. Further, in the absence of EV-delivered MMP14, the pulmonary fibrosis process deteriorated [155,156,157]. Macrophage-delivered exosomes also have a disintegrin and metalloproteinase, ADAM10, and ADAM15 on their surface and they cleave and activate TNFα, EGF, and collagen IV [157,158,159]. Further, ADAM10 can transactivate EGFR via G protein-coupled receptors, increasing the likelihood of the development of pathological settings in IPF [160]. Even though the ADAM family works on collagen degradation as MMPs [157,161], TGF-β also promotes ADAM10 expression and myofibroblast activation occurs via ADAM10-mediated sEphrin-B2 generation. Further, pharmacological inhibition of ADAM10 prevents lung fibrosis in mice [162]. Likewise, smoke increased ADAM15 expression in alveolar macrophages and in airway α-SMA-positive cells, while both ADAM10 and ADAM15 shed E-cadherin and changed the cell membrane structure [163,164,165]. Overall, the current studies show that macrophage-derived EVs are an important factor in establishing cell-to-cell communication and reorganizing the extracellular matrix via collagen degradation, cleavage of membrane proteins, stimulation of fibrotic signaling pathways, and activation of macrophages. Progression of fibrosis can be modulated by the different cargos in the EVs, therefore understanding their role in IPF may prove critical. 

### 3.5. Cell Senescence and Genetic Factors

IPF is an age-related disease. Indeed, cellular senescence, telomere shortening, epigenetic modifications, and mitochondrial dysfunction can contribute to the aging mechanisms in IPF [166,167,168,169,170]. During aging, phagocytotic cells start to lose their function, basal innate immune signaling is increased and the injury-related responses and cellular defense are decreased. As the macrophage phenotype shifts with ageing, coupled with enhanced oxidative metabolism, loss of migration capabilities, their basal release of inflammatory factors may impact their pathological imprint on the tissue [166,167,168,169,170]. Indeed, with aging, the alveolar microenvironment displays a constant inflammatory state, characterized by increased release of IFN-γ, TNF-α, IL-12, IL-1β, IL-6, and IL-10 by aged macrophages [171]. Moreover, senescence of the alveolar epithelial cell type 2 (ATII) increases IGF-1 expression, and polarization of M2 macrophages through IL-13 signaling [172]. On the other hand, plasminogen activator inhibitor 1-mediated TGF-β1-induced ATII cell senescence activates alveolar macrophages and contributes to lung fibrogenesis by inducing p16 [173]. Old macrophages can release more IL-10, a profibrotic cytokine, than young ones [174]. Macrophage migration inhibitory factor (MIF) is related to telomere shortening and ECM deposition, and it has been reported that MIF levels are higher in the fibrotic niches in the lung tissue and that MIF is increased in the BAL of IPF patients compared to controls [175,176]. Overall, IPF is an age-progressive disease and with the progressive ageing of the world population its incidence may increase in the years to come, making efforts to understand its pathological mechanisms even more crucial.

## 4. Macrophages as a Target

Targeting lung macrophages as a therapeutic might be useful for the treatment of fibrosis. Some studies show that blockade of the recruitment of the monocyte-derived macrophages in the fibrotic niche might attenuate the fibrotic processes [54,177,178]. A biomarker of lung transplantation/death CHI3L1 is produced by airway epithelial cells, alveolar type II epithelial cells, and macrophages in the lung, and the level of CHI3L1 is elevated in IPF patients [55,179]. It induces the M2 differentiation and their recruitment to the scar. Further, CHI3L1 recruits CD206^+^ mouse lung macrophages, which can stimulate fibroblast proliferation [55], making it an interesting therapeutic candidate and warranting more investigations.

Targeting macrophages at the cytokine level could be achieved via several pathways in addition to those mentioned in the previous sections. For example, serum amyloid P (SAP) targets collagen deposition via stimulating the release of IP10/CXCL10 by M2 macrophages and ameliorates bleomycin-induced pulmonary fibrosis [180,181]. SAP can control the peripheral blood monocyte differentiation and their activation states by binding to Fcγ receptors [180]. Another route could be via the targeting of the CCR4 signaling axis. Clinical and in vivo studies showed that CCR4 and its ligand CCL17 are regulators for fibrosis in the lung [182,183]. CCL17 led to M1 activation and oxidative injury by inducing NOS2. Further, in the absence of CCR4 in macrophages, the oxidative lung injury by bleomycin was decreased [183]. Likewise, the CCL2/CCR2 pathway are increased in the fibrotic niche and CCR2 deficiency decreases the fibrotic area through macrophage-derived MMP-2 and MMP-9 production [180,184]. Lastly, the Colony-Stimulating Factor Receptor-1 (CSF1R) is one of the signals that controls M1/M2 polarization towards M2. In the radiation-mediated pulmonary fibrosis model, investigators showed that activated IMs were able to induce myofibroblastic activation and ECM production via CSF1R activation, as treatment with the CSF1R mAb reversed the phenotype [58]. 

Targeting redox signaling and mitochondrial biogenesis in the lung macrophages might be another therapeutic approach. NOX4 is essential for macrophage polarization for fibrotic repair in the lung by inducing the production of profibrotic molecules for collagen deposition [178,185]. Further, Th2-independent, redox-dependent M2 polarization by regulation of STAT6 provides a potential therapeutic target for attenuating the progression of pulmonary fibrosis. STAT6 mediates Cu, Zn-SOD–induced M2 polarization in a redox-dependent manner by regulating the Jumonji domain containing (Jmjd) 3. Both STAT6 and Jmjd3 deficiency reduced the M2 polarization. Further, Leflunomide treatment reduced mitochondrial reactive oxygen species production and inhibited Jmjd3 expression and M2 polarization. Taken together, these observations provide evidence that the redox regulation of STAT6 and Jmjd3 is a unique regulatory mechanism for profibrotic M2 polarization [56,79].

Lung transplantation is the only current effective treatment for IPF. Although two antifibrotic drugs (Nintedanib and Pirfenidone) are approved and used for the treatment of IPF, they have side effects and they do not improve the survival rate [1,186]. Therefore, understanding the role of lung macrophage functions and using them as a potential therapeutic targets could prove critical. Pirfenidone inhibits pro-inflammatory cytokines (TNF-α, IL-1, IL-6, etc.) and promotes the production of anti-inflammatory cytokines (IL-10). However, this drug potentially targets fibroblasts and it can only delay the disease progression [187]. Macrophages express MMP2 and MMP9 that degrade collagen, and pirfenidone blocks the alternative activation of macrophages and modulates MMP activity [22,188]. Nintedanib blocks the polarization of both M1 and M2 human macrophages and markers that contribute to lung fibrosis (IL-1β, IL-8, IL-10, and CXCL13) by altering colony-stimulating factor 1 (CSF1) receptor (CSF1R) activation and its downstream PI3K/Akt signaling pathway [189]. 

Recently, the rescue and reprogramming of macrophages is becoming a novel therapeutic approach for the treatment of lung diseases, as highlighted by the treatment with the HIF-1α stabilizer, which promoted macrophage adaptation to the hypoxic microenvironment via their glycolytic metabolism, which resulted in the protection of the lungs from inflammation-induced injury [190]. To this end, several drugs have been shown to have an effect on the modulation of macrophage activity. Although not all of them have been used in IPF, their effect on macrophages could be relevant to pulmonary fibrosis. Indeed, some currently used treatments could interfere with the pathological fibrotic process, as detailed in Table 1, while other ones may exacerbate aberrant pathways (Table 2).

## 5. Conclusions

**Figure 2:**
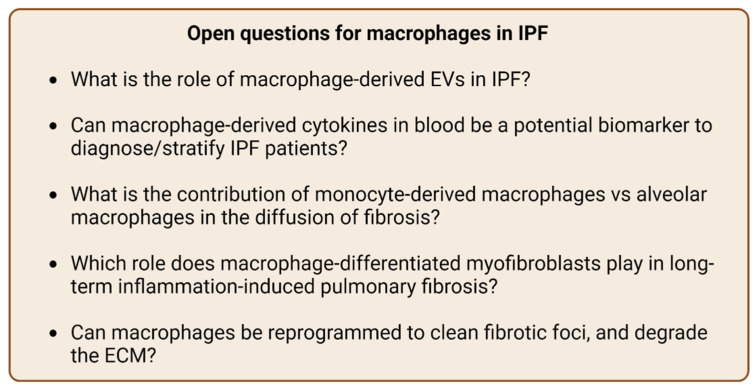
Open questions related to macrophage-related idiopathic pulmonary fibrosis.

In conclusion, macrophages promote collagen and ECM degradation, therefore reducing fibrosis, while on the other hand they can stimulate the fibrotic signaling pathways by inducing EMT, MTT, and Wnt signaling. IPF is a difficult disease to treat and there has been extensive research on this subject. In clinical studies, either there are insignificant results or the drugs may do more harm than good. Further, the pivotal role of macrophages on this disease is undeniable. However, more studies are needed to answer critical questions (Figure 2) leading to a better understanding of the mechanisms and inform more effective treatment strategies.

## Figures and Tables

**Table 1:** Therapeutics that could ameliorated the fibrotic process via macrophage activation.

Treatment	Mechanisms of Action	Effect on Macrophages
Atorvastatin	Lipid-decreasing statin	Reduces mediator production of AM (IL-1β, IL-6, and TNF-A-α) and macrophage recruitment [191,192]
Artemisinin	Antiviral, antimalarial, and anti-inflammatory	Inhibits macrophage chemotaxis and cytokine production [193]
Artesunate	Antimalarial	Attenuates proinflammatory effects of monocytes/macrophages [194]
Chloroquine	Immunosuppressive and Anti-parasite	Reduces TNF-A-α, IL-1-β and IL-6 [195,196]
Corticosteroid	Anti-inflammatory	Reduces macrophage CD64, CD80 and CD86 expression, controls the phenotype of alveolar macrophages [197].
Cyclophosphamide	Chemotherapy and Immunosuppressive	Activates and enhances macrophage phagocytosis [198]
Dasatinib	Chemotherapy	Elevates production of IL-10 while suppressing the production of IL-6, IL-12p40 and TNF-α in response to TLR stimulation [199]
HDAC6 Nexturastat A	HDAC inhibitor	Reduces pro-tumorigenic M2 macrophages [200].
Hydroxychloroquine	Immunosuppressive and Anti-parasite	Promotes apoptosis of macrophages and inhibits activation of macrophages, especially M2 macrophages [201]
Infliximab	Immunosuppressive	Induces apoptosis of Ly6C^+^ macrophages, decreases migration of monocytes into the ankles, and reduces CCL2 [202]
Intravenous Immunoglobulin (IVIG)	Therapy treatment for patients with antibody deficiencies	Inhibits the activation of monocytes and macrophages, inhibition of macrophage responses to IFN-γ [203]
PD-1/PD-L1 signaling blocker	Checkpoint inhibitor anticancer drug	Decreases TNF-A-α, IL-6, IFN-γ and ROS from alveolar macrophages [204]
Suberoylanilide hydroxamic acid (SAHA), Vorinostat	Chemotherapy	Reduces TNF-α, IL-1-β, IL-12, and IFN-γ [205]
Tocilizumab	Immunosuppressive	Anti-IL-6 receptor, modifies macrophage activation [206]
Zanubrutinib	Kinase inhibitor	Inhibits M1 macrophage polarization and promotes M2 macrophage polarization [207]

**Table 2:** Therapeutics that could exacerbate the fibrotic process via macrophage activation.

The Name of Drug	The Type of Drug	Effect on Macrophages
Amiodarone	Antiarrhythmic	Induces alveolar macrophages to secrete more TNF-α and superoxide anions [208,209].
Bleomycin	Chemotherapy	Recruits pro-fibrotic M2 cells and induces myofibroblast differentiation [31,66]
Cyclophosphamide	Chemotherapy and Immunosuppressive	Decreases spontaneous proliferation and reduces the ability to proliferate upon stimulation with GM-CSF [210]
Docetaxel	Chemotherapy	Induces M2 cells recruitment [211]
Methotrexate	Chemotherapy and Immunosuppressive drug	Macrophage recruitment [212]
Procainamide	Antiarrhythmic	Induces macrophage recruitment [213]

## Data Availability

Not applicable.

## Abbreviations

AMs	alveolar macrophages
ARDS	acute respiratory distress syndrome
ATII	alveolar epithelial cell type 2
BAL	bronchoalveolar lavage
CCR8	chemokine receptor 8
CDH11	cadherin-11
CSF1	colony-stimulating factor 1
CTGF	connective tissue growth factor
DAMPs	damage-associated molecular patterns
ECM	extracellular matrix
EGFR	epidermal growth factor receptor
EMT	Epithelial–mesenchymal transition
EVs	exosomes and extracellular vesicles
FGF-1	fibroblast growth factor receptor-1
IFIGENIA	controlled Idiopathic Pulmonary Fibrosis International Group Exploring N-Acetylcysteine I Annual
ILD	interstitial lung disease
IMs	interstitial macrophages
IPF	idiopathic pulmonary fibrosis
Jmjd	Jumonji domain containing
M1	classically activated macrophages
M2	alternatively activated macrophages
M-CSFR	colony-stimulating factor receptor
MFGE8	milk fat globule EGF 8
MIF	macrophage migration inhibitory factor
miRNA	microRNA
MMP	matrix metalloprotease
MMT	myofibroblast transition
Mo-AMs	monocyte-derived alveolar macrophages
mRNA	messenger RNA
NAC	N-acetylcysteine
PANTHER	Prednisone, Azathioprine, and N-Acetylcysteine: A Study That Evaluates Response
PAMPs	pathogen-associated molecular patterns
PDGF-R	platelet-derived growth factor receptor
PFT	pulmonary function test
RIF	radiation-induced lung fibrosis
rRNA	ribosomal RNA
SAP	serum amyloid P
TGF-β	transforming growth factor beta
TLR4	Toll-like receptor 4
TR-Mφ	tissue resident macrophages
tRNA	transfer RNA
UIP	usual interstitial pneumonia
VEGFR-2	vascular endothelial growth factor receptor-2

## References

[1] Kumar A., Kapnadak S.G., Girgis R.E., Raghu G. (2018). Lung transplantation in idiopathic pulmonary fibrosis. Expert Rev. Respir. Med..

[2] Raghu G., Collard H.R., Egan J.J., Martinez F.J., Behr J., Brown K.K., Colby T.V., Cordier J.-F., Flaherty K.R., Lasky J.A. (2011). An Official ATS/ERS/JRS/ALAT Statement: Idiopathic Pulmonary Fibrosis: Evidence-based Guidelines for Diagnosis and Management. Am. J. Respir. Crit. Care Med..

[3] Raghu G., Remy-Jardin M., Myers J.L., Richeldi L., Ryerson C.J., Lederer D.J., Behr J., Cottin V., Danoff S.K., Morell F. (2018). Diagnosis of Idiopathic Pulmonary Fibrosis. An Official ATS/ERS/JRS/ALAT Clinical Practice Guideline. Am. J. Respir. Crit. Care Med..

[4] Hutchinson J.P., McKeever T.M., Fogarty A.W., Navaratnam V., Hubbard R.B. (2014). Increasing Global Mortality from Idiopathic Pulmonary Fibrosis in the Twenty-First Century. Ann. Am. Thorac. Soc..

[5] Raghu G., Chen S.-Y., Yeh W.-S., Maroni B., Li Q., Lee Y.-C., Collard H.R. (2014). Idiopathic pulmonary fibrosis in US Medicare beneficiaries aged 65 years and older: Incidence, prevalence, and survival, 2001–2011. Lancet Respir. Med..

[6] Vancheri C., Failla M., Crimi N., Raghu G. (2010). Idiopathic pulmonary fibrosis: A disease with similarities and links to cancer biology. Eur. Respir. J..

[7] Raghu G., Remy-Jardin M., Richeldi L., Thomson C.C., Inoue Y., Johkoh T., Kreuter M., Lynch D.A., Maher T.M., Martinez F.J. (2022). Idiopathic Pulmonary Fibrosis (an Update) and Progressive Pulmonary Fibrosis in Adults: An Official ATS/ERS/JRS/ALAT Clinical Practice Guideline. Am. J. Respir. Crit. Care Med..

[8] Fernandez I.E., Eickelberg O. (2012). New cellular and molecular mechanisms of lung injury and fibrosis in idiopathic pulmonary fibrosis. Lancet.

[9] Jablonski R.P., Kim S., Cheresh P., Williams D.B., Morales-Nebreda L., Cheng Y., Yeldandi A., Bhorade S., Pardo A., Selman M. (2017). SIRT3 deficiency promotes lung fibrosis by augmenting alveolar epithelial cell mitochondrial DNA damage and apoptosis. FASEB J..

[10] Crosby L.M., Waters C.M. (2010). Epithelial repair mechanisms in the lung. Am. J. Physiol. Cell. Mol. Physiol..

[11] Shochet G.E., Brook E., Eyal O., Edelstein E., Shitrit D. (2019). Epidermal growth factor receptor paracrine upregulation in idiopathic pulmonary fibrosis fibroblasts is blocked by nintedanib. Am. J. Physiol. Cell. Mol. Physiol..

[12] Hewlett J.C., Kropski J.A., Blackwell T.S. (2018). Idiopathic pulmonary fibrosis: Epithelial-mesenchymal interactions and emerging therapeutic targets. Matrix Biol..

[13] Du Bois R.M., Wells A.U. (2001). Cryptogenic fibrosing alveolitis/idiopathic pulmonary fibrosis. Eur. Respir. J. Suppl..

[14] Rudd R.M., Haslam P.L., Turner-Warwick M. (1981). Cryptogenic fibrosing alveolitis. Relationships of pulmonary physiology and bronchoalveolar lavage to response to treatment and prognosis. Am. Rev. Respir. Dis..

[15] Raghu G., DePaso W.J., Cain K., Hammar S.P., Wetzel C.E., Dreis D.F., Hutchinson J., Pardee N.E., Winterbauer R.H. (1991). Azathioprine Combined with Prednisone in the Treatment of Idiopathic Pulmonary Fibrosis: A Prospective Double-blind, Randomized, Placebo-controlled Clinical Trial. Am. Rev. Respir. Dis..

[16] Behr J., Maier K., Degenkolb B., Krombach F., Vogelmeier C. (1997). Antioxidative and clinical effects of high-dose N-acetylcysteine in fibrosing alveolitis. Adjunctive therapy to maintenance immunosuppression. Am. J. Respir. Crit. Care Med..

[17] Demedts M., Behr J., Buhl R., Costabel U., Dekhuijzen R., Jansen H.M., MacNee W., Thomeer M., Wallaert B., Laurent F. (2005). High-Dose Acetylcysteine in Idiopathic Pulmonary Fibrosis. N. Engl. J. Med..

[18] Idiopathic Pulmonary Fibrosis Clinical Research N., Raghu G., Anstrom K.J., King T.E., Lasky J.A., Martinez F.J. (2012). Prednisone, azathioprine, and N-acetylcysteine for pulmonary fibrosis. N. Engl. J. Med..

[19] King T.E., Albera C., Bradford W.Z., Costabel U., Hormel P., Lancaster L., Noble P.W., Sahn S.A., Szwarcberg J., Thomeer M. (2009). Effect of interferon gamma-1b on survival in patients with idiopathic pulmonary fibrosis (INSPIRE): A multicentre, randomised, placebo-controlled trial. Lancet.

[20] Raghu G., Brown K.K., Bradford W.Z., Starko K., Noble P.W., Schwartz D.A., King T.E. (2004). A Placebo-Controlled Trial of Interferon Gamma-1b in Patients with Idiopathic Pulmonary Fibrosis. N. Engl. J. Med..

[21] King T.E., Bradford W.Z., Castro-Bernardini S., Fagan E.A., Glaspole I., Glassberg M.K., Gorina E., Hopkins P.M., Kardatzke D., Lancaster L. (2014). A Phase 3 Trial of Pirfenidone in Patients with Idiopathic Pulmonary Fibrosis. N. Engl. J. Med..

[22] Noble P.W., Albera C., Bradford W.Z., Costabel U., Glassberg M.K., Kardatzke D., King T.E., Lancaster L., Sahn S.A., Szwarcberg J. (2011). Pirfenidone in patients with idiopathic pulmonary fibrosis (CAPACITY): Two randomised trials. Lancet.

[23] Richeldi L., Du Bois R.M., Raghu G., Azuma A., Brown K.K., Costabel U., Cottin V., Flaherty K.R., Hansell D.M., Inoue Y. (2014). Efficacy and Safety of Nintedanib in Idiopathic Pulmonary Fibrosis. N. Engl. J. Med..

[24] Raghu G., Rochwerg B., Zhang Y., Cuello-Garcia C., Azuma A., Behr J., Brozek J.L., Collard H.R., Cunningham W., Homma S. (2015). An Official ATS/ERS/JRS/ALAT Clinical Practice Guideline: Treatment of Idiopathic Pulmonary Fibrosis. An Update of the 2011 Clinical Practice Guideline. Am. J. Respir. Crit. Care Med..

[25] Schaefer C.J., Ruhrmund D.W., Pan L., Seiwert S.D., Kossen K. (2011). Antifibrotic activities of pirfenidone in animal models. Eur. Respir. Rev..

[26] Wollin L., Wex E., Pautsch A., Schnapp G., Hostettler K., Stowasser S., Kolb M. (2015). Mode of action of nintedanib in the treatment of idiopathic pulmonary fibrosis. Eur. Respir. J..

[27] Distler O., Highland K.B., Gahlemann M., Azuma A., Fischer A., Mayes M.D., Raghu G., Sauter W., Girard M., Alves M. (2019). Nintedanib for Systemic Sclerosis–Associated Interstitial Lung Disease. N. Engl. J. Med..

[28] Flaherty K.R., Wells A.U., Cottin V., Devaraj A., Walsh S.L., Inoue Y., Richeldi L., Kolb M., Tetzlaff K., Stowasser S. (2019). Nintedanib in Progressive Fibrosing Interstitial Lung Diseases. N. Engl. J. Med..

[29] Wells A.U., Flaherty K.R., Brown K.K., Inoue Y., Devaraj A., Richeldi L., Moua T., Crestani B., Wuyts W.A., Stowasser S. (2020). Nintedanib in patients with progressive fibrosing interstitial lung diseases-subgroup analyses by interstitial lung disease diagnosis in the INBUILD trial: A randomised, double-blind, placebo-controlled, parallel-group trial. Lancet Respir. Med..

[30] Eapen M.S., Hansbro P.M., McAlinden K., Kim R.Y., Ward C., Hackett T.L., Walters E.H., Sohal S.S. (2017). Abnormal M1/M2 macrophage phenotype profiles in the small airway wall and lumen in smokers and chronic obstructive pulmonary disease (COPD). Sci. Rep..

[31] Hou J., Shi J., Chen L., Lv Z., Chen X., Cao H., Xiang Z., Han X. (2018). M2 macrophages promote myofibroblast differentiation of LR-MSCs and are associated with pulmonary fibrogenesis. Cell Commun. Signal..

[32] Mora A.L., Torres-González E., Rojas M., Corredor C., Ritzenthaler J., Xu J., Roman J., Brigham K., Stecenko A. (2006). Activation of Alveolar Macrophages via the Alternative Pathway in Herpesvirus-Induced Lung Fibrosis. Am. J. Respir. Cell Mol. Biol..

[33] Wahl S.M., Hunt D.A., Wakefield L.M., McCartney-Francis N., Wahl L.M., Roberts A.B., Sporn M.B. (1987). Transforming growth factor type beta induces monocyte chemotaxis and growth factor production. Proc. Natl. Acad. Sci. USA.

[34] Epelman S., LaVine K.J., Randolph G.J. (2014). Origin and Functions of Tissue Macrophages. Immunity.

[35] Gordon S. (2003). Alternative activation of macrophages. Nat. Rev. Immunol..

[36] Araki N., Hatae T., Furukawa A., Swanson J.A. (2003). Phosphoinositide-3-kinase-independent contractile activities associated with Fcγ-receptor-mediated phagocytosis and macropinocytosis in macrophages. J. Cell. Sci..

[37] Biswas S.K., Mantovani A. (2010). Macrophage plasticity and interaction with lymphocyte subsets: Cancer as a paradigm. Nat. Immunol..

[38] Gregory C.D., Devitt A. (2004). The macrophage and the apoptotic cell: An innate immune interaction viewed simplistically?. Immunology.

[39] Desai O., Winkler J., Minasyan M., Herzog E.L. (2018). The Role of Immune and Inflammatory Cells in Idiopathic Pulmonary Fibrosis. Front. Med..

[40] Van Furth R. (1976). Macrophage activity and clinical immunology. Origin and kinetics of mononuclear phagocytes. Ann. N. Y. Acad. Sci..

[41] van Furth R., Cohn Z.A. (1968). The origin and kinetics of mononuclear phagocytes. J. Exp. Med..

[42] Bowden D.H., Adamson I.Y. (1972). The pulmonary interstitial cell as immediate precursor of the alveolar macrophage. Am. J. Pathol..

[43] Bedoret D., Wallemacq H., Marichal T., Desmet C., Quesada Calvo F., Henry E., Closset R., Dewals B.G., Thielen C., Gustin P. (2009). Lung interstitial macrophages alter dendritic cell functions to prevent airway allergy in mice. J. Clin. Investig..

[44] Cai Y., Sugimoto C., Arainga M., Alvarez-Hernandez X., Didier E., Kuroda M.J. (2014). In Vivo Characterization of Alveolar and Interstitial Lung Macrophages in Rhesus Macaques: Implications for Understanding Lung Disease in Humans. J. Immunol..

[45] Franke-Ullmann G., Pförtner C., Walter P., Steinmüller C., Lohmann-Matthes M.L., Kobzik L. (1996). Characterization of murine lung interstitial macrophages in comparison with alveolar macrophages in vitro. J. Immunol..

[46] Martin T.R., Frevert C.W. (2005). Innate immunity in the lungs. Proc. Am. Thorac. Soc..

[47] Schneberger D., Aharonson-Raz K., Singh B. (2011). Monocyte and macrophage heterogeneity and Toll-like receptors in the lung. Cell Tissue Res..

[48] Puttur F., Gregory L.G., Lloyd C.M. (2019). Airway macrophages as the guardians of tissue repair in the lung. Immunol. Cell Biol..

[49] Tan S., Krasnow M.A. (2016). Developmental origin of lung macrophage diversity. Development.

[50] Mantovani A., Sica A., Sozzani S., Allavena P., Vecchi A., Locati M. (2004). The chemokine system in diverse forms of macrophage activation and polarization. Trends Immunol..

[51] Mills C.D., Kincaid K., Alt J.M., Heilman M.J., Hill A.M. (2000). M-1/M-2 macrophages and the Th1/Th2 paradigm. J. Immunol..

[52] Mantovani A., Sozzani S., Locati M., Allavena P., Sica A. (2002). Macrophage polarization: Tumor-associated macrophages as a paradigm for polarized M2 mononuclear phagocytes. Trends Immunol..

[53] Martinez F.O., Gordon S. (2014). The M1 and M2 paradigm of macrophage activation: Time for reassessment. F1000Prime Rep..

[54] Misharin A.V., Morales-Nebreda L., Reyfman P.A., Cuda C.M., Walter J.M., McQuattie-Pimentel A.C., Chen C.-I., Anekalla K.R., Joshi N., Williams K.J.N. (2017). Monocyte-derived alveolar macrophages drive lung fibrosis and persist in the lung over the life span. J. Exp. Med..

[55] Zhou Y., Peng H., Sun H., Peng X., Tang C., Gan Y., Chen X., Mathur A., Hu B., Slade M.D. (2014). Chitinase 3–Like 1 Suppresses Injury and Promotes Fibroproliferative Responses in Mammalian Lung Fibrosis. Sci. Transl. Med..

[56] He C., Larson-Casey J.L., Gu L., Ryan A.J., Murthy S., Carter A.B. (2016). Cu,Zn–Superoxide Dismutase–Mediated Redox Regulation of Jumonji Domain Containing 3 Modulates Macrophage Polarization and Pulmonary Fibrosis. Am. J. Respir. Cell Mol. Biol..

[57] Murthy S., Larson-Casey J.L., Ryan A.J., He C., Kobzik L., Carter A.B. (2015). Alternative activation of macrophages and pulmonary fibrosis are modulated by scavenger receptor, macrophage receptor with collagenous structure. FASEB J..

[58] Meziani L., Mondini M., Petit B., Boissonnas A., De Montpreville V.T., Mercier O., Vozenin M.-C., Deutsch E. (2018). CSF1R inhibition prevents radiation pulmonary fibrosis by depletion of interstitial macrophages. Eur. Respir. J..

[59] Joshi N., Watanabe S., Verma R., Jablonski R.P., Chen C.-I., Cheresh P., Markov N., Reyfman P.A., McQuattie-Pimentel A.C., Sichizya L. (2020). A spatially restricted fibrotic niche in pulmonary fibrosis is sustained by M-CSF/M-CSFR signalling in monocyte-derived alveolar macrophages. Eur. Respir. J..

[60] Ji W.-J., Ma Y.-Q., Zhou X., Zhang Y.-D., Lu R.-Y., Sun H.-Y., Guo Z.-Z., Zhang Z., Li Y.-M., Wei L.-Q. (2014). Temporal and spatial characterization of mononuclear phagocytes in circulating, lung alveolar and interstitial compartments in a mouse model of bleomycin-induced pulmonary injury. J. Immunol. Methods.

[61] Naik P.N., Horowitz J.C., Moore T.A., Wilke C.A., Toews G.B., Moore B.B. (2012). Pulmonary fibrosis induced by γ-herpesvirus in aged mice is associated with increased fibroblast responsiveness to transforming growth factor-β. J. Gerontol. A Biol. Sci. Med. Sci..

[62] Byrne A.J., Mathie S.A., Gregory L.G., Lloyd C.M. (2015). Pulmonary macrophages: Key players in the innate defence of the airways. Thorax.

[63] Barleon B., Sozzani S., Zhou D., Weich H.A., Mantovani A., Marme D. (1996). Migration of human monocytes in response to vascular endothelial growth factor (VEGF) is mediated via the VEGF receptor flt-1. Blood.

[64] Tian L., Yu Q., Liu D., Chen Z., Zhang Y., Lu J., Ma X., Huang F., Han J., Wei L. (2022). Epithelial–mesenchymal Transition of Peritoneal Mesothelial Cells Is Enhanced by M2c Macrophage Polarization. Immunol. Investig..

[65] Pakshir P., Alizadehgiashi M., Wong B., Coelho N.M., Chen X., Gong Z., Shenoy V.B., McCulloch C.A., Hinz B. (2019). Dynamic fibroblast contractions attract remote macrophages in fibrillar collagen matrix. Nat. Commun..

[66] Ballinger M.N., Newstead M.W., Zeng X., Bhan U., Mo X.M., Kunkel S.L., Moore B.B., Flavell R., Christman J.W., Standiford T.J. (2015). IRAK-M Promotes Alternative Macrophage Activation and Fibroproliferation in Bleomycin-Induced Lung Injury. J. Immunol..

[67] Srivastava M., Saqib U., Naim A., Roy A., Liu D., Bhatnagar D., Ravinder R., Baig M.S. (2017). The TLR4-NOS1-AP1 signaling axis regulates macrophage polarization. Inflamm. Res..

[68] Bolourani S., Sari E., Brenner M., Wang P. (2021). Extracellular CIRP Induces an Inflammatory Phenotype in Pulmonary Fibroblasts via TLR4. Front. Immunol..

[69] Papiris S.A., Tomos I.P., Karakatsani A., Spathis A., Korbila I., Analitis A., Kolilekas L., Kagouridis K., Loukides S., Karakitsos P. (2018). High levels of IL-6 and IL-8 characterize early-on idiopathic pulmonary fibrosis acute exacerbations. Cytokine.

[70] Pechkovsky D.V., Prasse A., Kollert F., Engel K.M., Dentler J., Luttmann W., Friedrich K., Müller-Quernheim J., Zissel G. (2010). Alternatively activated alveolar macrophages in pulmonary fibrosis—Mediator production and intracellular signal transduction. Clin. Immunol..

[71] Thorley A.J., Ford P.A., Giembycz M.A., Goldstraw P., Young A., Tetley T.D. (2007). Differential Regulation of Cytokine Release and Leukocyte Migration by Lipopolysaccharide-Stimulated Primary Human Lung Alveolar Type II Epithelial Cells and Macrophages. J. Immunol..

[72] Fitzner N., Clauberg S., Essmann F., Liebmann J., Kolb-Bachofen V. (2008). Human Skin Endothelial Cells Can Express All 10 *TLR* Genes and Respond to Respective Ligands. Clin. Vaccine Immunol..

[73] Pinhal-Enfield G., Ramanathan M., Hasko G., Vogel S.N., Salzman A.L., Boons G.-J., Leibovich S.J. (2003). An Angiogenic Switch in Macrophages Involving Synergy between Toll-Like Receptors 2, 4, 7, and 9 and Adenosine A2A Receptors. Am. J. Pathol..

[74] Smiley S.T., King J.A., Hancock W.W. (2001). Fibrinogen Stimulates Macrophage Chemokine Secretion Through Toll-Like Receptor 4. J. Immunol..

[75] Srikrishna G., Freeze H.H. (2009). Endogenous Damage-Associated Molecular Pattern Molecules at the Crossroads of Inflammation and Cancer. Neoplasia.

[76] Swanson L., Katkar G.D., Tam J., Pranadinata R.F., Chareddy Y., Coates J., Anandachar M.S., Castillo V., Olson J., Nizet V. (2020). TLR4 signaling and macrophage inflammatory responses are dampened by GIV/Girdin. Proc. Natl. Acad. Sci. USA.

[77] Barbarin V., Xing Z., Delos M., Lison M., Huaux F. (2005). Pulmonary overexpression of IL-10 augments lung fibrosis and Th2 responses induced by silica particles. Am. J. Physiol. Cell. Mol. Physiol..

[78] Büttner C., Skupin A., Reimann T., Rieber E.P., Unteregger G., Geyer P., Frank K.-H. (1997). Local Production of Interleukin-4 During Radiation-induced Pneumonitis and Pulmonary Fibrosis in Rats: Macrophages as a Prominent Source of Interleukin-4. Am. J. Respir. Cell Mol. Biol..

[79] He C., Ryan A.J., Murthy S., Carter A.B. (2013). Accelerated Development of Pulmonary Fibrosis via Cu,Zn-superoxide Dismutase-induced Alternative Activation of Macrophages. J. Biol. Chem..

[80] García-Fojeda B., Minutti C.M., Montero-Fernández C., Stamme C., Casals C. (2022). Signaling Pathways That Mediate Alveolar Macrophage Activation by Surfactant Protein A and IL-4. Front. Immunol..

[81] Fichtner-Feigl S., Strober W., Kawakami K., Puri R.K., Kitani A. (2006). IL-13 signaling through the IL-13α_2_ receptor is involved in induction of TGF-β_1_ production and fibrosis. Nat. Med..

[82] Kaviratne M., Hesse M., Leusink M., Cheever A.W., Davies S.J., McKerrow J.H., Wakefield L.M., Letterio J.J., Wynn T.A. (2004). IL-13 activates a mechanism of tissue fibrosis that is completely TGF-β independent. J. Immunol..

[83] Lee C.G., Homer R.J., Zhu Z., Lanone S., Wang X., Koteliansky V., Shipley J.M., Gotwals P., Noble P., Chen Q. (2001). Interleukin-13 induces tissue fibrosis by selectively stimulating and activating transforming growth factor β_1_. J. Exp. Med..

[84] Heldin C.H., Miyazono K., ten Dijke P. (1997). TGF-β signalling from cell membrane to nucleus through SMAD proteins. Nature.

[85] Sari E., Oztay F., Tasci A.E. (2020). Vitamin D modulates E-cadherin turnover by regulating TGF-β and Wnt signalings during EMT-mediated myofibroblast differentiation in A459 cells. J. Steroid Biochem. Mol. Biol..

[86] Letterio J.J., Roberts A.B. (1998). Regulation of immune responses by TGF-β. Annu. Rev. Immunol..

[87] Miettinen P.J., Ebner R., Lopez A.R., Derynck R. (1994). TGF-β induced transdifferentiation of mammary epithelial cells to mesenchymal cells: Involvement of type I receptors. J. Cell Biol..

[88] Roberts A.B., Anzano M.A., Wakefield L.M., Roche N.S., Stern D.F., Sporn M.B. (1985). Type beta transforming growth factor: A bifunctional regulator of cellular growth. Proc. Natl. Acad. Sci. USA.

[89] Roberts A.B., Sporn M.B., Assoian R.K., Smith J.M., Roche N.S., Wakefield L.M., Heine U.I., Liotta L.A., Falanga V., Kehrl J.H. (1986). Transforming growth factor type beta: Rapid induction of fibrosis and angiogenesis in vivo and stimulation of collagen formation in vitro. Proc. Natl. Acad. Sci. USA.

[90] Wipff P.J., Rifkin D.B., Meister J.J., Hinz B. (2007). Myofibroblast contraction activates latent TGF-β1 from the extracellular matrix. J. Cell Biol..

[91] Yildirim M., Kayalar O., Atahan E., Oztay F. (2022). Atorvastatin attenuates pulmonary fibrosis in mice and human lung fibroblasts, by the regulation of myofibroblast differentiation and apoptosis. J. Biochem. Mol. Toxicol..

[92] Yildirim M., Oztay F., Kayalar O., Tasci A.E. (2021). Effect of long noncoding RNAs on epithelial-mesenchymal transition in A549 cells and fibrotic human lungs. J. Cell. Biochem..

[93] Branton M.H., Kopp J.B. (1999). TGF-β and fibrosis. Microbes Infect..

[94] Coker R.K., Laurent G.J., Shahzeidi S., Lympany P.A., Du Bois R.M., Jeffery P.K., McAnulty R.J. (1997). Transforming growth factors-β1, -β2, and -β3 stimulate fibroblast procollagen production in vitro but are differentially expressed during bleomycin-induced lung fibrosis. Am. J. Pathol..

[95] Zhang F., Wang H., Wang X., Jiang G., Liu H., Zhang G., Wang H., Fang R., Bu X., Cai S. (2016). TGF-β induces M2-like macrophage polarization via SNAIL-mediated suppression of a pro-inflammatory phenotype. Oncotarget.

[96] Yu X., Buttgereit A., Lelios I., Utz S.G., Cansever D., Becher B., Greter M. (2017). The Cytokine TGF-β Promotes the Development and Homeostasis of Alveolar Macrophages. Immunity.

[97] Königshoff M., Balsara N., Pfaff E.-M., Kramer M., Chrobak I., Seeger W., Eickelberg O. (2008). Functional Wnt Signaling Is Increased in Idiopathic Pulmonary Fibrosis. PLoS ONE.

[98] Morrisey E.E. (2003). Wnt Signaling and Pulmonary Fibrosis. Am. J. Pathol..

[99] Lam A.P., Herazo-Maya J.D., Sennello J.A., Flozak A.S., Russell S., Mutlu G.M., Budinger G.R.S., DasGupta R., Varga J., Kaminski N. (2014). Wnt Coreceptor *Lrp5* Is a Driver of Idiopathic Pulmonary Fibrosis. Am. J. Respir. Crit. Care Med..

[100] Zhu L., Fu X., Chen X., Han X., Dong P. (2017). M2 macrophages induce EMT through the TGF-β/Smad2 signaling pathway. Cell Biol. Int..

[101] Murray L.A., Chen Q., Kramer M.S., Hesson D.P., Argentieri R.L., Peng X., Gulati M., Homer R.J., Russell T., van Rooijen N. (2011). TGF-β driven lung fibrosis is macrophage dependent and blocked by Serum amyloid P. Int. J. Biochem. Cell Biol..

[102] Borthwick L., Barron L.D., Hart K.M., Vannella K.M., Thompson R.W., Oland S., Cheever A.W., Sciurba J., Ramalingam T.R., Fisher A.J. (2016). Macrophages are critical to the maintenance of IL-13-dependent lung inflammation and fibrosis. Mucosal Immunol..

[103] Hesketh M., Sahin K.B., West Z.E., Murray R.Z. (2017). Macrophage Phenotypes Regulate Scar Formation and Chronic Wound Healing. Int. J. Mol. Sci..

[104] Nacu N., Luzina I.G., Highsmith K., Lockatell V., Pochetuhen K., Cooper Z.A., Gillmeister M.P., Todd N.W., Atamas S.P. (2008). Macrophages produce TGF-β-induced (β-ig-h3) following ingestion of apoptotic cells and regulate MMP14 levels and collagen turnover in fibroblasts. J. Immunol..

[105] Hay E.D. (2005). The mesenchymal cell, its role in the embryo, and the remarkable signaling mechanisms that create it. Dev. Dyn..

[106] Moustakas A., Heldin C.-H. (2007). Signaling networks guiding epithelial-mesenchymal transitions during embryogenesis and cancer progression. Cancer Sci..

[107] Willis B.C., Borok Z. (2007). TGF-β-induced EMT: Mechanisms and implications for fibrotic lung disease. Am. J. Physiol. Lung Cell. Mol. Physiol..

[108] Miyazono K. (2009). Transforming growth factor-β signaling in epithelial-mesenchymal transition and progression of cancer. Proc. Jpn. Acad. Ser. B.

[109] Tan T.K., Zheng G., Hsu T.-T., Lee S.R., Zhang J., Zhao Y., Tian X., Wang Y., Wang Y.M., Cao Q. (2013). Matrix metalloproteinase-9 of tubular and macrophage origin contributes to the pathogenesis of renal fibrosis via macrophage recruitment through osteopontin cleavage. Lab. Investig..

[110] Bednarczyk R.B., Tuli N.Y., Hanly E.K., Ben Rahoma G., Maniyar R., Mittelman A., Geliebter J., Tiwari R.K. (2018). Macrophage inflammatory factors promote epithelial-mesenchymal transition in breast cancer. Oncotarget.

[111] Macias-Ceja D.C., Coll S., Bauset C., Seco-Cervera M., Gisbert-Ferrandiz L., Navarro F., Cosin-Roger J., Calatayud S., Barrachina M.D., Ortiz-Masia D. (2022). IFNγ-Treated Macrophages Induce EMT through the WNT Pathway: Relevance in Crohn’s Disease. Biomedicines.

[112] Fu X.T., Dai Z., Song K., Zhang Z.J., Zhou Z.J., Zhou S.L., Zhao Y.M., Xiao Y.S., Sun Q.M., Ding Z.B. (2015). Macrophage-secreted IL-8 induces epithelial-mesenchymal transition in hepatocellular carcinoma cells by activating the JAK2/STAT3/Snail pathway. Int. J. Oncol..

[113] An M., Li D., Yuan M., Li Q., Zhang L., Wang G. (2017). Different macrophages equally induce EMT in endometria of adenomyosis and normal. Reproduction.

[114] Shi J., Li Q., Sheng M., Zheng M., Yu M., Zhang L. (2016). The Role of TLR4 in M1 Macrophage-Induced Epithelial-Mesenchymal Transition of Peritoneal Mesothelial Cells. Cell. Physiol. Biochem..

[115] Hu Y., He M.-Y., Zhu L.-F., Yang C.-C., Zhou M.-L., Wang Q., Zhang W., Zheng Y.-Y., Wang D.-M., Xu Z.-Q. (2016). Tumor-associated macrophages correlate with the clinicopathological features and poor outcomes via inducing epithelial to mesenchymal transition in oral squamous cell carcinoma. J. Exp. Clin. Cancer Res..

[116] Li S., Xu F., Zhang J., Wang L., Zheng Y., Wu X., Wang J., Huang Q., Lai M. (2018). Tumor-associated macrophages remodeling EMT and predicting survival in colorectal carcinoma. OncoImmunology.

[117] Hesse M., Modolell M., La Flamme A.C., Schito M., Fuentes J.M., Cheever A.W., Pearce E.J., Wynn T.A. (2001). Differential regulation of nitric oxide synthase-2 and arginase-1 by type 1/type 2 cytokines in vivo: Granulomatous pathology is shaped by the pattern of L-arginine metabolism. J. Immunol..

[118] Song E., Ouyang N., Hörbelt M., Antus B., Wang M., Exton M.S. (2000). Influence of Alternatively and Classically Activated Macrophages on Fibrogenic Activities of Human Fibroblasts. Cell. Immunol..

[119] Vierhout M., Ayoub A., Naiel S., Yazdanshenas P., Revill S.D., Reihani A., Dvorkin-Gheva A., Shi W., Ask K. (2021). Monocyte and macrophage derived myofibroblasts: Is it fate? A review of the current evidence. Wound Repair Regen..

[120] Shook B.A., Wasko R.R., Rivera-Gonzalez G.C., Salazar-Gatzimas E., López-Giráldez F., Dash B.C., Muñoz-Rojas A.R., Aultman K.D., Zwick R.K., Lei V. (2018). Myofibroblast proliferation and heterogeneity are supported by macrophages during skin repair. Science.

[121] Lodyga M., Cambridge E., Karvonen H.M., Pakshir P., Wu B., Boo S., Kiebalo M., Kaarteenaho R., Glogauer M., Kapoor M. (2019). Cadherin-11-mediated adhesion of macrophages to myofibroblasts establishes a profibrotic niche of active TGF-β. Sci. Signal..

[122] Bhandari R., Ball M.S., Martyanov V., Popovich D., Schaafsma E., Han S., ElTanbouly M., Orzechowski N.M., Carns M., Arroyo E. (2020). Profibrotic Activation of Human Macrophages in Systemic Sclerosis. Arthritis Rheumatol..

[123] Liu T., Jin H., Ullenbruch M., Hu B., Hashimoto N., Moore B., McKenzie A., Lukacs N.W., Phan S.H. (2004). Regulation of found in inflammatory zone 1 expression in bleomycin-induced lung fibrosis: Role of IL-4/IL-13 and mediation via STAT-6. J. Immunol..

[124] Vasse G.F., Nizamoglu M., Heijink I.H., Schleputz M., van Rijn P., Thomas M.J., Burgess J.K., Melgert B.N. (2021). Macrophage-stroma interactions in fibrosis: Biochemical, biophysical, and cellular perspectives. J. Pathol..

[125] Little K., Llorián-Salvador M., Tang M., Du X., Marry S., Chen M., Xu H. (2020). Macrophage to myofibroblast transition contributes to subretinal fibrosis secondary to neovascular age-related macular degeneration. J. Neuroinflamm..

[126] Meng X.-M., Wang S., Huang X.-R., Yang C., Xiao J., Zhang Y., To K.-F., Nikolic-Paterson D., Lan H.-Y. (2016). Inflammatory macrophages can transdifferentiate into myofibroblasts during renal fibrosis. Cell Death Dis..

[127] Wang S., Meng X.M., Ng Y.Y., Ma F.Y., Zhou S., Zhang Y., Yang C., Huang X.R., Xiao J., Wang Y.Y. (2016). TGF-β/Smad3 signalling regulates the transition of bone marrow-derived macrophages into myofibroblasts during tissue fibrosis. Oncotarget.

[128] Wang Y.-Y., Jiang H., Pan J., Huang X.-R., Wang Y.-C., Huang H.-F., To K.-F., Nikolic-Paterson D.J., Lan H.-Y., Chen J.-H. (2017). Macrophage-to-Myofibroblast Transition Contributes to Interstitial Fibrosis in Chronic Renal Allograft Injury. J. Am. Soc. Nephrol..

[129] Cui H., Xie N., Banerjee S., Ge J., Jiang D., Dey T., Mathews Q., Liu R.-M., Liu G. (2021). Lung Myofibroblast Promotes Macrophage Pro-Fibrotic Activity through Lactate Induced Histone Lactylation. Am. J. Respir. Cell Mol. Biol..

[130] Bonnans C., Chou J., Werb Z. (2014). Remodelling the extracellular matrix in development and disease. Nat. Rev. Mol. Cell Biol..

[131] Aimes R.T., Quigley J.P. (1995). Matrix metalloproteinase-2 is an interstitial collagenase. Inhibitor-free enzyme catalyzes the cleavage of collagen fibrils and soluble native type I collagen generating the specific 3/4- and 1/4-length fragments. J. Biol. Chem..

[132] Ohuchi E., Imai K., Fujii Y., Sato H., Seiki M., Okada Y. (1997). Membrane Type 1 Matrix Metalloproteinase Digests Interstitial Collagens and Other Extracellular Matrix Macromolecules. J. Biol. Chem..

[133] Zhao X., Chen J., Sun H., Zhang Y., Zou D. (2022). New insights into fibrosis from the ECM degradation perspective: The macrophage-MMP-ECM interaction. Cell Biosci..

[134] Cabrera S., Gaxiola M., Arreola J.L., Ramírez R., Jara P., D’Armiento J., Richards T., Selman M., Pardo A. (2007). Overexpression of MMP9 in macrophages attenuates pulmonary fibrosis induced by bleomycin. Int. J. Biochem. Cell Biol..

[135] Eickelberg O., Kohler E., Reichenberger F., Bertschin S., Woodtli T., Erne P., Perruchoud A.P., Roth M. (1999). Extracellular matrix deposition by primary human lung fibroblasts in response to TGF-β1 and TGF-β3. Am. J. Physiol..

[136] Fujishima S., Shiomi T., Yamashita S., Yogo Y., Nakano Y., Inoue T., Nakamura M., Tasaka S., Hasegawa N., Aikawa N. (2010). Production and activation of matrix metalloproteinase 7 (matrilysin 1) in the lungs of patients with idiopathic pulmonary fibrosis. Arch. Pathol. Lab. Med..

[137] Gharib S.A., Johnston L.K., Huizar I., Birkland T.P., Hanson J., Wang Y., Parks W.C., Manicone A.M. (2014). MMP28 promotes macrophage polarization toward M2 cells and augments pulmonary fibrosis. J. Leukoc. Biol..

[138] Madala S.K., Pesce J.T., Ramalingam T.R., Wilson M.S., Minnicozzi S., Cheever A.W., Thompson R.W., Mentink-Kane M.M., Wynn T.A. (2010). Matrix Metalloproteinase 12-Deficiency Augments Extracellular Matrix Degrading Metalloproteinases and Attenuates IL-13–Dependent Fibrosis. J. Immunol..

[139] Atabai K., Jame S., Azhar N., Kuo A., Lam M., McKleroy W., DeHart G., Rahman S., Xia D.D., Melton A.C. (2009). Mfge8 diminishes the severity of tissue fibrosis in mice by binding and targeting collagen for uptake by macrophages. J. Clin. Investig..

[140] Van Niel G., Porto-Carreiro I., Simoes S., Raposo G. (2006). Exosomes: A Common Pathway for a Specialized Function. J. Biochem..

[141] Raposo G., Nijman H.W., Stoorvogel W., Liejendekker R., Harding C.V., Melief C.J., Geuze H.J. (1996). B lymphocytes secrete antigen-presenting vesicles. J. Exp. Med..

[142] Witwer K.W., Buzás E.I., Bemis L.T., Bora A., Lässer C., Lötvall J., Nolte-’t Hoen E.N., Piper M.G., Sivaraman S., Skog J. (2013). Standardization of sample collection, isolation and analysis methods in extracellular vesicle research. J. Extracell. Vesicles.

[143] Artlett C.M., Sassi-Gaha S., Hope J.L., Feghali-Bostwick C.A., Katsikis P.D. (2017). Mir-155 is overexpressed in systemic sclerosis fibroblasts and is required for NLRP3 inflammasome-mediated collagen synthesis during fibrosis. Arthritis Res. Ther..

[144] Liu G., Friggeri A., Yang Y., Milosevic J., Ding Q., Thannickal V.J., Kaminski N., Abraham E. (2010). miR-21 mediates fibrogenic activation of pulmonary fibroblasts and lung fibrosis. J. Exp. Med..

[145] Santos-Álvarez J.C., Velázquez-Enríquez J.M., García-Carrillo R., Rodríguez-Beas C., Ramírez-Hernández A.A., Reyes-Jiménez E., González-García K., López-Martínez A., Mayoral L.P.-C., Aguilar-Ruiz S.R. (2022). miRNAs Contained in Extracellular Vesicles Cargo Contribute to the Progression of Idiopathic Pulmonary Fibrosis: An In Vitro Aproach. Cells.

[146] Aston C., Jagirdar J., Lee T.C., Hur T., Hintz R.L., Rom W. (1995). Enhanced insulin-like growth factor molecules in idiopathic pulmonary fibrosis. Am. J. Respir. Crit. Care Med..

[147] Elliot S., Periera-Simon S., Xia X., Catanuto P., Rubio G., Shahzeidi S., El Salem F., Shapiro J., Briegel K., Korach K.S. (2019). MicroRNA let-7 Downregulates Ligand-Independent Estrogen Receptor–mediated Male-Predominant Pulmonary Fibrosis. Am. J. Respir. Crit. Care Med..

[148] Banerjee S., Xie N., Cui H., Tan Z., Yang S., Icyuz M., Abraham E., Liu G. (2013). MicroRNA let-7c Regulates Macrophage Polarization. J. Immunol..

[149] Su S., Zhao Q., He C., Huang D., Liu J., Chen F., Chen J., Liao J.-Y., Cui X., Zeng Y. (2015). miR-142-5p and miR-130a-3p are regulated by IL-4 and IL-13 and control profibrogenic macrophage program. Nat. Commun..

[150] Kishore A., Petrek M. (2021). Roles of Macrophage Polarization and Macrophage-Derived miRNAs in Pulmonary Fibrosis. Front. Immunol..

[151] Wang Y., Wang X., Zhang H., Han B., Ye Y., Zhang M., Wang Y., Xue J., Wang C. (2021). Transforming Growth Factor-β1 Promotes M1 Alveolar Macrophage Polarization in Acute Lung Injury by Up-Regulating DNMT1 to Mediate the microRNA-124/PELI1/IRF5 Axis. Front. Cell. Infect. Microbiol..

[152] Li X., Yang N., Cheng Q., Zhang H., Liu F., Shang Y. (2021). MiR-21-5p in Macrophage-Derived Exosomes Targets Smad7 to Promote Epithelial Mesenchymal Transition of Airway Epithelial Cells. J. Asthma Allergy.

[153] Bandari S.K., Purushothaman A., Ramani V.C., Brinkley G.J., Chandrashekar D.S., Varambally S., Mobley J.A., Zhang Y., Brown E.E., Vlodavsky I. (2018). Chemotherapy induces secretion of exosomes loaded with heparanase that degrades extracellular matrix and impacts tumor and host cell behavior. Matrix Biol..

[154] Hakulinen J., Sankkila L., Sugiyama N., Lehti K., Keski-Oja J. (2008). Secretion of active membrane type 1 matrix metalloproteinase (MMP-14) into extracellular space in microvesicular exosomes. J. Cell. Biochem..

[155] Placido L., Romero Y., Maldonado M., Toscano-Marquez F., Ramírez R., Calyeca J., Mora A., Selman M., Pardo A. (2021). Loss of MT1-MMP in Alveolar Epithelial Cells Exacerbates Pulmonary Fibrosis. Int. J. Mol. Sci..

[156] Sanderson R.D., Bandari S.K., Vlodavsky I. (2019). Proteases and glycosidases on the surface of exosomes: Newly discovered mechanisms for extracellular remodeling. Matrix Biol..

[157] Shimoda M., Khokha R. (2013). Proteolytic factors in exosomes. Proteomics.

[158] Duffy M.J., Mullooly M., O’Donovan N., Sukor S., Crown J., Pierce A., McGowan P.M. (2011). The ADAMs family of proteases: New biomarkers and therapeutic targets for cancer?. Clin. Proteom..

[159] Lee H.D., Koo B., Kim Y.H., Jeon O., Kim D. (2012). Exosome release of ADAM15 and the functional implications of human macrophage-derived ADAM15 exosomes. FASEB J..

[160] Yan Y., Shirakabe K., Werb Z. (2002). The metalloprotease Kuzbanian (ADAM10) mediates the transactivation of EGF receptor by G protein–coupled receptors. J. Cell Biol..

[161] Solomon J.B. (1966). Induction of antibody formation to goat erythrocytes in the developing chick embryo and effects of maternal antibody. Immunology.

[162] Lagares D., Ghassemi-Kakroodi P., Tremblay C., Santos A., Probst C.K., Franklin A., Santos D.M., Grasberger P., Ahluwalia N., Montesi S.B. (2017). ADAM10-mediated ephrin-B2 shedding promotes myofibroblast activation and organ fibrosis. Nat. Med..

[163] Maretzky T., Reiss K., Ludwig A., Buchholz J., Scholz F., Proksch E., de Strooper B., Hartmann D., Saftig P. (2005). ADAM10 mediates E-cadherin shedding and regulates epithelial cell-cell adhesion, migration, and β-catenin translocation. Proc. Natl. Acad. Sci. USA.

[164] Najy A.J., Day K.C., Day M.L. (2008). The Ectodomain Shedding of E-cadherin by ADAM15 Supports ErbB Receptor Activation. J. Biol. Chem..

[165] Wang X., Zhang D., Higham A., Wolosianka S., Gai X., Zhou L., Petersen H., Pinto-Plata V., Divo M., Silverman E.K. (2020). ADAM15 expression is increased in lung CD8^+^ T cells, macrophages, and bronchial epithelial cells in patients with COPD and is inversely related to airflow obstruction. Respir. Res..

[166] Alder J.K., Chen J.J., Lancaster L., Danoff S., Su S.C., Cogan J.D., Vulto I., Xie M., Qi X., Tuder R.M. (2008). Short telomeres are a risk factor for idiopathic pulmonary fibrosis. Proc. Natl. Acad. Sci. USA.

[167] Hecker L., Logsdon N.J., Kurundkar D., Kurundkar A., Bernard K., Hock T., Meldrum E., Sanders Y.Y., Thannickal V.J. (2014). Reversal of Persistent Fibrosis in Aging by Targeting Nox4-Nrf2 Redox Imbalance. Sci. Transl. Med..

[168] Minagawa S., Araya J., Numata T., Nojiri S., Hara H., Yumino Y., Kawaishi M., Odaka M., Morikawa T., Nishimura S.L. (2011). Accelerated epithelial cell senescence in IPF and the inhibitory role of SIRT6 in TGF-β-induced senescence of human bronchial epithelial cells. Am. J. Physiol. Lung Cell. Mol. Physiol..

[169] Tsakiri K.D., Cronkhite J.T., Kuan P.J., Xing C., Raghu G., Weissler J.C., Rosenblatt R.L., Shay J.W., Garcia C.K. (2007). Adult-onset pulmonary fibrosis caused by mutations in telomerase. Proc. Natl. Acad. Sci. USA.

[170] Faner R., Rojas M., MacNee W., Agustí A. (2012). Abnormal Lung Aging in Chronic Obstructive Pulmonary Disease and Idiopathic Pulmonary Fibrosis. Am. J. Respir. Crit. Care Med..

[171] Canan C.H., Gokhale N.S., Carruthers B., Lafuse W.P., Schlesinger L.S., Torrelles J.B., Turner J. (2014). Characterization of lung inflammation and its impact on macrophage function in aging. J. Leukoc. Biol..

[172] Chung E.J., Kwon S., Reedy J.L., White A.O., Song J.S., Hwang I., Chung J.Y., Ylaya K., Hewitt S.M., Citrin D.E. (2021). IGF-1 Receptor Signaling Regulates Type II Pneumocyte Senescence and Resulting Macrophage Polarization in Lung Fibrosis. Int. J. Radiat. Oncol..

[173] Rana T., Jiang C., Liu G., Miyata T., Antony V., Thannickal V.J., Liu R.M. (2020). PAI-1 Regulation of TGF-β1-induced Alveolar Type II Cell Senescence, SASP Secretion, and SASP-mediated Activation of Alveolar Macrophages. Am. J. Respir. Cell Mol. Biol..

[174] Kelly J., Khan A.A., Yin J., Ferguson T.A., Apte R.S. (2007). Senescence regulates macrophage activation and angiogenic fate at sites of tissue injury in mice. J. Clin. Investig..

[175] Bargagli E., Olivieri C., Nikiforakis N., Cintorino M., Magi B., Perari M., Vagaggini C., Spina D., Prasse A., Rottoli P. (2009). Analysis of macrophage migration inhibitory factor (MIF) in patients with idiopathic pulmonary fibrosis. Respir. Physiol. Neurobiol..

[176] Mathew B., Jacobson J.R., Siegler J.H., Moitra J., Blasco M., Xie L., Unzueta C., Zhou T., Evenoski C., Al-Sakka M. (2013). Role of Migratory Inhibition Factor in Age-Related Susceptibility to Radiation Lung Injury via NF-E2–Related Factor–2 and Antioxidant Regulation. Am. J. Respir. Cell Mol. Biol..

[177] Gibbons M.A., MacKinnon A.C., Ramachandran P., Dhaliwal K., Duffin R., Phythian-Adams A.T., van Rooijen N., Haslett C., Howie S.E., Simpson A.J. (2011). Ly6C^hi^ Monocytes Direct Alternatively Activated Profibrotic Macrophage Regulation of Lung Fibrosis. Am. J. Respir. Crit. Care Med..

[178] He C., Larson-Casey J.L., Davis D., Hanumanthu V.S., Longhini A.L.F., Thannickal V.J., Gu L., Carter A.B. (2019). NOX4 modulates macrophage phenotype and mitochondrial biogenesis in asbestosis. JCI Insight.

[179] Korthagen N.M., van Moorsel C.H., Barlo N.P., Ruven H.J., Kruit A., Heron M., Bosch J.M.V.D., Grutters J.C. (2011). Serum and BALF YKL-40 levels are predictors of survival in idiopathic pulmonary fibrosis. Respir. Med..

[180] Murray L.A., Rosada R., Moreira A.P., Joshi A., Kramer M.S., Hesson D.P., Argentieri R.L., Mathai S., Gulati I., Herzog E.L. (2010). Serum Amyloid P Therapeutically Attenuates Murine Bleomycin-Induced Pulmonary Fibrosis via Its Effects on Macrophages. PLoS ONE.

[181] Pilling D., Roife D., Wang M., Ronkainen S.D., Crawford J.R., Travis E.L., Gomer R. (2007). Reduction of Bleomycin-Induced Pulmonary Fibrosis by Serum Amyloid P. J. Immunol..

[182] Pignatti P., Brunetti G., Moretto D., Yacoub M.-R., Fiori M., Balbi B., Balestrino A., Cervio G., Nava S., Moscato G. (2006). Role of the Chemokine Receptors CXCR3 and CCR4 in Human Pulmonary Fibrosis. Am. J. Respir. Crit. Care Med..

[183] Trujillo G., O’Connor E.C., Kunkel S.L., Hogaboam C.M. (2008). A Novel Mechanism for CCR4 in the Regulation of Macrophage Activation in Bleomycin-Induced Pulmonary Fibrosis. Am. J. Pathol..

[184] Okuma T., Terasaki Y., Kaikita K., Kobayashi H., Kuziel W.A., Kawasuji M., Takeya M. (2004). C-C chemokine receptor 2 (CCR2) deficiency improves bleomycin-induced pulmonary fibrosis by attenuation of both macrophage infiltration and production of macrophage-derived matrix metalloproteinases. J. Pathol..

[185] He C., Carter A.B. (2015). The Metabolic Prospective and Redox Regulation of Macrophage Polarization. J. Clin. Cell. Immunol..

[186] Myllarniemi M., Kaarteenaho R. (2015). Pharmacological treatment of idiopathic pulmonary fibrosis—Preclinical and clinical studies of pirfenidone, nintedanib, and N-acetylcysteine. Eur. Clin. Respir. J..

[187] Lederer D.J., Martinez F.J. (2018). Idiopathic Pulmonary Fibrosis. N. Engl. J. Med..

[188] Toda M., Mizuguchi S., Minamiyama Y., Yamamoto-Oka H., Aota T., Kubo S., Nishiyama N., Shibata T., Takemura S. (2018). Pirfenidone suppresses polarization to M2 phenotype macrophages and the fibrogenic activity of rat lung fibroblasts. J. Clin. Biochem. Nutr..

[189] Bellamri N., Morzadec C., Joannes A., Lecureur V., Wollin L., Jouneau S., Vernhet L. (2019). Alteration of human macrophage phenotypes by the anti-fibrotic drug nintedanib. Int. Immunopharmacol..

[190] Woods P.S., Kimmig L.M., Sun K.A., Meliton A.Y., Shamaa O.R., Tian Y., Cetin-Atalay R., Sharp W.W., Hamanaka R.B., Mutlu G.M. (2022). HIF-1α induces glycolytic reprograming in tissue-resident alveolar macrophages to promote cell survival during acute lung injury. Elife.

[191] Melo A.C., Cattani-Cavalieri I., Barroso M.V., Quesnot N., Gitirana L.B., Lanzetti M., Valença S.S. (2018). Atorvastatin dose-dependently promotes mouse lung repair after emphysema induced by elastase. Biomed. Pharmacother..

[192] Sakamoto N., Hayashi S., Mukae H., Vincent R., Hogg J.C., van Eeden S.F. (2009). Effect of Atorvastatin on PM_10_-induced Cytokine Production by Human Alveolar Macrophages and Bronchial Epithelial Cells. Int. J. Toxicol..

[193] Morad H.O.J., Luqman S., Pinto L.G., Cunningham K.P., Vilar B., Clayton G., Shankar-Hari M., McNaughton P.A. (2022). Artemisinin inhibits neutrophil and macrophage chemotaxis, cytokine production and NET release. Sci. Rep..

[194] Konkimalla B., Blunder M., Korn B., Soomro S.A., Jansen H., Chang W., Posner G.H., Bauer R., Efferth T. (2008). Effect of artemisinins and other endoperoxides on nitric oxide-related signaling pathway in RAW 264.7 mouse macrophage cells. Nitric Oxide.

[195] Jang C.H., Choi J.H., Byun M.S., Jue D.M. (2006). Chloroquine inhibits production of TNF-α, IL-1β and IL-6 from lipopolysaccharide-stimulated human monocytes/macrophages by different modes. Rheumatology.

[196] Silva R., Tan L., Rodrigues D.A., Prestes E.B., Gomes C.P., Gama A.M., Oliveira P.L., Paiva C.N., Manoury B., Bozza M.T. (2021). Chloroquine inhibits pro-inflammatory effects of heme on macrophages and invivo. Free Radic. Biol. Med..

[197] Higham A., Scott T., Li J., Gaskell R., Dikwa A.B., Shah R., Montero-Fernandez M.A., Lea S., Singh D. (2020). Effects of corticosteroids on COPD lung macrophage phenotype and function. Clin. Sci..

[198] Roghanian A., Hu G., Fraser C., Singh M., Foxall R.B., Meyer M.J., Lees E., Huet H., Glennie M.J., Beers S.A. (2019). Cyclophosphamide Enhances Cancer Antibody Immunotherapy in the Resistant Bone Marrow Niche by Modulating Macrophage FcγR Expression. Cancer Immunol. Res..

[199] Ozanne J., Prescott A.R., Clark K. (2015). The clinically approved drugs dasatinib and bosutinib induce anti-inflammatory macrophages by inhibiting the salt-inducible kinases. Biochem. J..

[200] Knox T., Sahakian E., Banik D., Hadley M., Palmer E., Noonepalle S., Kim J., Powers J., Gracia-Hernandez M., Oliveira V. (2019). Selective HDAC6 inhibitors improve anti-PD-1 immune checkpoint blockade therapy by decreasing the anti-inflammatory phenotype of macrophages and down-regulation of immunosuppressive proteins in tumor cells. Sci. Rep..

[201] Zheng H., Zhang Y., He J., Yang Z., Zhang R., Li L., Luo Z., Ye Y., Sun Q. (2021). Hydroxychloroquine Inhibits Macrophage Activation and Attenuates Renal Fibrosis After Ischemia-Reperfusion Injury. Front. Immunol..

[202] Huang Q.-Q., Birkett R., Doyle R., Shi B., Roberts E.L., Mao Q., Pope R.M. (2018). The Role of Macrophages in the Response to TNF Inhibition in Experimental Arthritis. J. Immunol..

[203] Park-Min K.H., Serbina N.V., Yang W., Ma X., Krystal G., Neel B.G., Nutt S.L., Hu X., Ivashkiv L.B. (2007). FcγRIII-dependent inhibition of interferon-γ responses mediates suppressive effects of intravenous immune globulin. Immunity.

[204] Hu J.F., Zhang W., Zuo W., Tan H.Q., Bai W. (2020). Inhibition of the PD-1/PD-L1 signaling pathway enhances innate immune response of alveolar macrophages to mycobacterium tuberculosis in mice. Pulm. Pharmacol. Ther..

[205] Leoni F., Zaliani A., Bertolini G., Porro G., Pagani P., Pozzi P., Donà G., Fossati G., Sozzani S., Azam T. (2002). The antitumor histone deacetylase inhibitor suberoylanilide hydroxamic acid exhibits antiinflammatory properties via suppression of cytokines. Proc. Natl. Acad. Sci. USA.

[206] Shimizu M., Mizuta M., Okamoto N., Yasumi T., Iwata N., Umebayashi H., Okura Y., Kinjo N., Kubota T., Nakagishi Y. (2020). Tocilizumab modifies clinical and laboratory features of macrophage activation syndrome complicating systemic juvenile idiopathic arthritis. Pediatr. Rheumatol..

[207] Li X., Wei Y., Li S., Liang J., Liu Z., Cui Y., Gao J., Yang Z., Li L., Zhou H. (2022). Zanubrutinib ameliorates lipopolysaccharide-induced acute lung injury via regulating macrophage polarization. Int. Immunopharmacol..

[208] El-Mohandes E.M., Moustafa A.M., Khalaf H.A., Hassan Y.F. (2017). The role of mast cells and macrophages in amiodarone induced pulmonary fibrosis and the possible attenuating role of atorvastatin. Biotech. Histochem..

[209] Zitnik R.J., Cooper J.A., Rankin J.A., Sussman J. (1992). Effects of in vitro amiodarone exposure on alveolar macrophage inflammatory mediator production. Am. J. Med. Sci..

[210] Santosuosso M., Divangahi M., Zganiacz A., Xing Z. (2002). Reduced tissue macrophage population in the lung by anticancer agent cyclophosphamide: Restoration by local granulocyte macrophage–colony-stimulating factor gene transfer. Blood.

[211] Guan W., Hu J., Yang L., Tan P., Tang Z., West B.L., Bollag G., Xu H., Wu L. (2019). Inhibition of TAMs improves the response to docetaxel in castration-resistant prostate cancer. Endocr. Relat. Cancer.

[212] Imokawa S., Colby T., Leslie K., Helmers R. (2000). Methotrexate pneumonitis: Review of the literature and histopathological findings in nine patients. Eur. Respir. J..

[213] Thangam M., Nathan S., Petrovica M., Kar B., Patel M., Loyalka P., Buja L.M., Gregoric I.D. (2015). Procainamide-induced pulmonary fibrosis after orthotopic heart transplantation: A case report and literature review. Cardiovasc. Pathol..

